# Validation and characterization of DNA microarray gene expression data distribution and associated moments

**DOI:** 10.1186/1471-2105-11-576

**Published:** 2010-11-24

**Authors:** Reuben Thomas, Luis de la Torre, Xiaoqing Chang, Sanjay Mehrotra

**Affiliations:** 1 Environmental Systems Biology Group, Laboratory of Molecular Toxicology, National Institute of Environmental Health Sciences, RTP, NC 27709 USA; 2 Department of Industrial Engineering and Management Sciences, Northwestern University, Evanston, IL USA

## Abstract

**Background:**

The data from DNA microarrays are increasingly being used in order to understand effects of different conditions, exposures or diseases on the modulation of the expression of various genes in a biological system. This knowledge is then further used in order to generate molecular mechanistic hypotheses for an organism when it is exposed to different conditions. Several different methods have been proposed to analyze these data under different distributional assumptions on gene expression. However, the empirical validation of these assumptions is lacking.

**Results:**

Best fit hypotheses tests, moment-ratio diagrams and relationships between the different moments of the distribution of the gene expression was used to characterize the observed distributions. The data are obtained from the publicly available gene expression database, Gene Expression Omnibus (GEO) to characterize the empirical distributions of gene expressions obtained under varying experimental situations each of which providing relatively large number of samples for hypothesis testing. All data were obtained from either of two microarray platforms - the commercial Affymetrix mouse 430.2 platform and a non-commercial Rosetta/Merck one. The data from each platform were preprocessed in the same manner.

**Conclusions:**

The null hypotheses for goodness of fit for all considered univariate theoretical probability distributions (including the Normal distribution) are rejected for more than 50% of probe sets on the Affymetrix microarray platform at a 95% confidence level, suggesting that under the tested conditions *a priori *assumption of any of these distributions across all probe sets is not valid. The pattern of null hypotheses rejection was different for the data from Rosetta/Merck platform with only around 20% of the probe sets failing the logistic distribution goodness-of-fit test. We find that there are statistically significant (at 95% confidence level based on the F-test for the fitted linear model) relationships between the mean and the logarithm of the coefficient of variation of the distributions of the logarithm of gene expressions. An additional novel statistically significant quadratic relationship between the skewness and kurtosis is identified. Data from both microarray platforms fail to identify with any one of the chosen theoretical probability distributions from an analysis of the l-moment ratio diagram.

## Background

The current biological literature makes extensive use of gene mRNA expression data from experimental systems called gene chips/gene micro-arrays. These data are used to infer genomic level conclusions. For example, to infer the response of an organism or cell culture under treatment or perturbation. Microarrays as an experimental system are very valuable in that they provide a genome-wide picture (for all the (~30000) genes). Unfortunately, because of costs of collecting microarray data, the number of samples per treatment is quite small (~2-10).

The data from microarrays are noisy. There are a number of reasons to expect variability in the measurements of the expressions of the genes in mammalian organisms. These include biological causes, or the noise associated with the steps involved in the measurement of the gene expression. Depending on the question that the researcher is trying to answer, he/she would have to control for many of these sources of variation of gene expression. This paper is interested in understanding the variation in the expression data after the known/reported sources of variation have been controlled for.

The biological variability could be due to genetic or non-genetic factors [1] studies the cis-acting variation that explains differences of 15 genes in the human brain [2] reviews literature to suggest that the allele-specific differences in the rates of transcription are common. Other studies demonstrating the influence of genotype on gene expression include [3] (in the human thyroid tissue), [4,5] (in human lymphoblastoid cell lines), [6] (human blood leukocytes), [7] (in human liver tissue) and [8] (in liver tissue from mice of different strains).

Among the non-genetic factors explaining the variation in gene expression include the gender of the organism - [9] (in human skeletal muscle), [10] (in human white blood cells), [11] (in human peripheral blood mononuclear cells), [12] (in human retina), [13] (in human blood) and [14] (in the liver and kidney of mice and rats). Age of the organism was shown to be a significant covariate in [12] (in human retina), [11] (in human peripheral blood mononuclear cells), [9] (in human skeletal muscle) and [13] (in human blood). There is a significant variability of gene expression across different tissue or cell types [15,16]. There is even variation within a given tissue due to the presence of multiple cell types - [11,13] (in human blood), [9] (in human skeletal muscle) and [17] (in human placenta). Other important factors include the diet and fasting status of the organism [14] and the time of day that the samples were taken [13]. Another important variability factor is the environmental condition that the organism was under before the tissue sample was taken - for example the mice could be sleep deprived [18], undergoing craniofacial development [19], given oral doses of synthetic triglycerides [20] or fed doses of chemicals that are known to be lung carcinogens [21] or it could be medical students under psychological stress before a major exam [22].

The other classes of non-genetic gene expression variability that has been studied intensively (theoretically and experimentally) have been termed intrinsic, extrinsic and pathway-specific or global noises (see [23,24] for reviews on this). Intrinsic noise is assigned to variation arising because of the stochastic nature of transcription and translation due to the small number of mRNA and protein molecules. Extrinsic noise could be due to changes in the cellular environment. These noises can be demonstrated experimentally by observing the output of two different reporters for the same gene in the same cell and separate cells. Pathway-specific noise can be viewed as the noise that is transferred along all the genes whose genes sequentially participate in given cellular or biochemical process.

Variability could arise at various stages involved in getting the output from a microarray and also after data from the microarray are obtained. This is in terms of intensity measurements (for the predefined surrogates for different genes that are termed probe sets) being normalized and preprocessed to get estimates of gene expressions. RNA are isolated from the cells obtained from the tissue sample has been drawn from the organism. The RNA are then subjected to the process of reverse transcription (RT) to obtain cDNA that are then subjected to the vitro transcription (IVT) process to obtain cRNA using polymerases. The cRNA are then hybridized to probes on the microarray platform [25]. The factor that influence the final intensity measurements for different probes include the amount of polymerase used for the IVT process, the amount of time allotted for this process by the experimenter and the binding specificity of the cRNA to the corresponding probe sequence on the microarray platform [26]. Tu et al [25] performed a small controlled analysis of the noise characteristics in the gene expression data from microarrays. They provide empirical distributions of gene expressions arising just because of the variation introduced by the above described process of getting to intensity values from the microarray, i.e., they performed an analysis of the measurement variability. It does not seem trivial to control for a consistent microarray experiment protocol - [14] demonstrated that the laboratory where the microarray experiment was performed is a major source of variation [27] found that microarray experiments performed in different years had different characteristics of gene expression. Different microarray data preprocessing and normalization algorithms generate expression data with different characteristics [28]. In addition, microarrays as tools for detecting changes in gene expression have been shown to be sensitive and specific in a well defined range of its output or gene expression. Low gene expression can be confused with optical noise while due to saturation, changes at higher levels of gene expression are more difficult to detect [29].

Most of the current journals require the microarray data to be deposited on a database (like the Gene Expression Omnibus, GEO [30]) if these data were used for analysis in a paper. The data deposited on the database are in the form of multiple samples corresponding to each of multiple conditions (typically one of these conditions would correspond to the normal situation when there is no disease or toxin dosed). The data are deposited depending on the type of microarray chip used. There are different commercial manufactures of microarrays - Affymetrix and Agilent being the most popular. Each of these manufacturers makes chips for specific organisms (mouse, rat, frog, human zebrafish etc.) and within chips for specific organisms also there are different varieties.

One of the main areas where microarray data has had its application is in the identification of differentially expressed genes across varying treatment conditions. The approaches used could be classified based on whether they use parametric assumptions about the underlying distribution the gene expression or not. Kerr et al [31] used a ANOVA model to capture variation of gene expression arising from two color microarrays. The non-normality of the residuals of this model was noted in this work. Newton et al [32] assume that all gene expressions are gamma distributed - a distribution that is right skewed and whose coefficient of variation decreases with increasing mean. They attempted to validate this using real data and found that the fits did capture the primary features of variation but were poor in general. Rocke and Durbin [33] assume a additive-multiplicative error model for gene expression that is additive at low levels of gene expression and multiplicative at higher levels of expression. So by this one would expect the gene expression to be log-normally distributed at high levels of gene expression. Then there are a class of approaches called empirical Bayes methods - [34-36] are examples - that make prior assumptions about the distribution of gene expression and then use the data itself to get the posterior probability of the gene being differentially expressed. They are all based on the observation that mean and the coefficient of variation of the distribution of gene expression have a definite pattern of variation (eg. see [37]). The typical microarray set up uses a relatively small number of samples to generate data for tens of thousands of genes. The empirical Bayes approaches essentially attempt to improve the estimation of the variance of a given gene by utilizing the observed pattern of variation between mean and the coefficient of variation of expression of all the target genes in the system [34,35] assume normal priors of gene expression [36] assumes a normal prior for the difference in means of the gene expression over the two conditions. Non-parametric methods have also been proposed to be used for the analysis of differential expression (eg. see [38]). Popular permutation-based method based on a modified t statistic is the so called SAM statistic and its modifications [39,40]. Bayesian network inference represents another analysis that makes use of gene expression data and typically assumes that the data is normally distributed - [41] and [42]. An additional analysis where gene expression has been significantly used is in the development of a prediction model or a classifier - for example in the development of a classifier between two different forms of leukemia [43], prediction of prognosis of patients with breast cancer [44] and the prediction of potential carcinogenicity of a chemical [45]. Such analyses do not make distributional assumptions on the expression of the gene expression data and the efficacy of the models developed in the above examples was done using cross-validation of data.

The above paragraph describes a snapshot of the analysis done using gene expression data, some of the analyses make use of distributional assumptions and some do not. Since distributional assumptions are made frequently, it appears prudent to validate this assumption. As mentioned above Newton et al [32] and Kerr et al [31] attempted to verify their distributional assumptions and did not find their assumptions adequately supported by the data. Tu et al [25] did not consider the analysis of variation due to biological variability. The literature thus lacks an empirical validation of the distribution of gene expression as measured by microarrays across multiple univariate theoretical distributions with sufficient amount of data.

Since 2002 a significant amount of data from sources like the Gene Expression Omnibus GEO [30] and ArrayExpress ([46] has become available. This data can now potentially be used to validate the distribution and noise assumptions for statistical analysis and to develop improved inference methods to analyze the microarray data.

This paper focuses on identifying and validating empirical distribution fits of genome-wide gene expressions as measured by microarrays. In addition to the normal distribution we empirically tested the empirical fit for a number of well established probability distributions.

We analyzed four microarray data sets from the GEO database [30]. They were all generated using the Affymetrix Mouse 430.2 platform. They are data from three tissues - brain, liver and craniofacial tissue and expression data for the so called "house keeping" genes [47] across over 6000 samples collected over a wide range of conditions. These data sets were chosen in part because they each had a relatively large number of samples generated by the same laboratory. Also, in light of the points mentioned the samples in two of the datasets (from the craniofacial and liver) were partially matched for gender, age, mouse strain tissue source and environmental conditions prior to sample collection. The data set from the brain involved three different strains of mice. The microarray data were preprocessed and normalized in the same manner. It is therefore expected that the primary sources of variability in gene expression would be a convolution of intrinsic, extrinsic, pathway-specific or global noise and noise associated with microarray sample preparation and hybridization to the DNA chip for the two data sets that each used the same strain of mice for all the samples. The brain data set would be expected to show additional genetic variation due to the utilization of three different strains in the generation of the samples.

## Methods

### Data sets used in the analysis

The microarray samples used in the analyses in this manuscript were based in part on six separate data sets (see Table 1 and Additional files 1, 2, 3, 4, Table S8, Table S3, Table S1 and Table S2 for better detail of the data in Table 1) obtained from the GEO database [30]. Four of them were from the Affymetrix Mouse Genome 430.2 array that uses a single color, in situ oligonucleotide array technology while the other two were from a non-commercial platform using a two-color, spotted oligonucleotide array technology.

**Table 1:** Description of the data sets used.

Data set	GEO accession	Technology	No. of Samples	Tissue	Data
*Craniofacial*	GSE7759	in situ oligonucleotide	105	craniofacial	Logarithm of transcript measure

*Liver*	GSE8396	in situ oligonucleotide	93	liver	Logarithm of transcript measure

*Brain*	GSE9444	in situ oligonucleotide	69	brain	Logarithm of transcript measure

*Housekeeping*	Additional file 4, Table S2	in situ oligonucleotide	6219	mixed	Logarithm of transcript measure

*Male*	GSE2814	spotted oligonucleotide	155	liver	Logarithm of transcript measure relative to common pool

*Female*	GSE2814	spotted oligonucleotide	156	liver	Logarithm of transcript measure relative to common pool

Further detailed description of these data sets are provided in Additional files 1, 2, 3, 4: Table S8, Table S3, Table S1 and Table S2

The GEO series codes for three of the Affymetrix data are GSE7759, GSE8396 and GSE9444-termed respectively as "Craniofacial", "Liver" and "Brain" based on the tissues from which the samples were drawn. The specific samples identified by their GSM codes that were used from the three data sets are provided in Additional file 3, Table S1. There were 105 samples present in the "Craniofacial" data set, 93 samples in the "Liver" data set and 69 samples in the "Brain" data set.

The data in the "Craniofacial" data set was analyzed in [19] where craniofacial development in mice was studied. C57BL/6J mice were sampled at 12 hour intervals from E10.5-E12.5. It is during this time that the facial structures in the mice form. Tissues from three distinct regions - frontonasal, maxillary and the mandibular prominence at each of these time points and analyzed for transcriptomic changes. Seven biological replicate samples were collected from each of these regions at every 12 hour time interval.

The data in the "Liver" data set was analyzed in [20] where the effects of various synthetic dietary triglycerides on the hepatic gene expression were studied. One of the objectives of this study was to understand the role of PPARα in the observed effects. Male mice of SV129 strain and PPARα-/- mice (2-6 months of age) on SV129 background were used. Apart from a control, the mice were treated with two synthetic agonists and four synthetic triglycerides. There were four or five biological replicate samples for each treatment and wild type or knockout mice combination.

The data in the "Brain" data set was analyzed in [18]. The objective of this study was to analyze the transcriptomic changes in the brain and the liver resulting from varying amounts of sleep loss in 3 different strains (AKR/J, DBA/2J and C57BL/6J) of mice. In this manuscript only a subset of the samples from the brain were used. The mice of each strain were deprived of sleep for various time periods. There were three biological replicate samples per combination of strain and time period of sleep deprivation.

In addition, 6219 microarray samples on the Affymetrix 430.2 platform were also downloaded from GEO [30]. The GSM sample ids along with a description of the experimental conditions under which the samples were collected are given in Additional file 4, Table S2. The samples were chosen using the GPLBrowse program [48] in April 2009. This program plots various moments of different samples on the GEO database versus each other. The scatter plot of the logarithm of mean of expression of all the samples versus the logarithm of the standard deviation of the samples on the GEO database from the Affymetrix Mouse 430.2 was visually examined. Samples that did not seem like outliers were chosen to form the set of samples that formed the basis of our analysis. Note that the raw data was downloaded in all cases and then preprocessed in a well-defined manner (see below). These samples were collected from mice of different strains, development stages, tissues, sex, and different laboratories over the past few years. The distribution of expression of the 21 house keeping genes identified in [47] was analyzed using this data.

The data from the non-commercial spotted oligonucleotide array (GEO accession: GSE2814) were analyzed in a number of papers [49-54] where one of the goals was to sex-specific and tissue-specific differences in gene expression. The data were from the liver of ApoE null (C57BL/6J × C3H/HeJ)F2 intercross mice. There were 155 male samples and 156 female samples - the data sets is hereafter called "Male" and "Female" respectively.

### Data preprocessing and normalization

For each of the four Affymetrix data sets used, raw CEL format data from GEO was normalized using the R Bioconductor [55] implementation of the GCRMA normalization procedure [56]. The Affymetrix Mouse 430.2 platform has data for 45101 probe sets in all. Each of the probe sets potentially map to a gene in the mouse genome. The samples in each of the "Craniofacial", "Liver" and "Brain" data sets were normalized separately. As described in the previous section, only a small subset of samples in each of three datasets was replicates, the remaining was obtained under different conditions (e.g. different development stage in a different facial region for the "Craniofacial" data). The altered conditions would have effects on the expressions of some of the genes in each of the data sets. In this manuscript, for each of the three datasets ("Craniofacial", "Liver" and "Brain") an attempt is made to identify those probe sets or genes whose expressions were not altered under all the conditions involved. This is done using the non-parametric Kruskal-Wallis one way analysis of variance test [57] for the expressions of each gene across all samples in a given data set. Probe sets with a p-value greater than a liberal 0.1 cutoff from the Kruskal Wallis test were deemed to be unaffected by any of the conditions involved in the generation of a given data set. Results of varying this 0.1 cutoff sensitivity to the results of distributional tests are provided in the Additional material. The Kruskal-Wallis test also assumes that the distributional form of the data being compared across the conditions remains the same. An alternate method that does not need the above assumption is the analysis using bootstrapping of residuals [58] from a heteroscedastic one-way anova model [59]. However, this assumption results in over 99% of all probe sets (data not shown) in each of the three data sets being deemed unchanged over conditions involved in generation of the probe sets.

For the data set of 6219 microarray samples RAM memory limitations prohibit normalizing all samples together. To normalize these, the following steps were followed:

1. The samples were partitioned into sets of 75.

2. The gene expression data for each of these 75 samples were obtained after running the GCRMA routine.

3. Using the data from step 2, the gene expression across the whole data set of 6219 samples were normalized with respect to each other using the quantile normalization method as described in(Bolstad B: Probe level quantile normalization of high density oligonucleotide array data. *Unpublished manuscript *2001.).

Yang et al [54] that use the "Male" and "Female" data define "active expressed" genes and a procedure to obtain them. In summary, genes differential expressed with respect to the reference pool are identified by a p-value cutoff of 0.01 from an error model used in their analysis. In addition to this set of transcriptionally active genes, genes that were significantly correlated (Pearson correlation p-value < 10^-5^) with these active genes were also chosen. This combined set of genes is what is used in the analysis in this paper. In the interest of having a relatively large number of genes for analysis, further criteria used by Yang et al [54] like presence of an eQTL with LOD score cutoff or significant correlations with the characterized traits of adiposity, plasma lipids, or atherosclerosis were ignored. The log ratio data were used then used as-is as downloaded from GEO.

### Testing Distributional Assumptions

For each of the data sets "Craniofacial", "Liver" and "Brain", "Housekeeping", "Male" and "Female", Kolmogorov-Smirnov (KS) and Anderson-Darling (AD) hypothesis tests were used to test distributional assumptions. Both test the null hypothesis that a set of data comes from a given distribution, with distributional parameters possibly unknown. The AD test is more sensitive to differences in the tails of the data than the KS distribution. In testing for Normality, the AD test is known to be more powerful than the KS test [60]. Normal, log-normal, logistic, log-logistic, Weibull, and extreme value distributions were tested. The "Male" and "Female" data were just tested on the normal, logistic and extreme value distributions because the log-ratio data from these data sets could be negative. The distributions were chosen because both tables of critical values for the tests with unknown distribution parameters and efficient implementations of parameter are available. In this manuscript the results for the AD are provided while those for the KS tests are provided in the Additional material.

For each of the data sets and each distribution *F*, each of identified (either by the Kruskal Wallis test for the "Craniofacial", "Liver" and "Brain" data sets or as actively expressed for the "Male" and "Female" data sets, as explained in the previous section) probe sets were tested in the following manner:

1. Use maximum likelihood estimation (MLE) to estimate $\overset{\wedge }{\theta }$ for *F*

2. Use the KS and AD tests at the 90% and 95% to test whether the probe data comes from $F(\overset{\wedge }{\theta })$.

For each of these distributions, MATLAB version R2009B Statistics Toolbox MLE functions were used. Critical values can be found in [60].

In addition, for housekeeping genes, KS and AD tests were used to test for gamma and Pareto distributions across a set of 6219 samples. For these distributions, tables of critical values do not exist, but a method for generating p-values for KS tests with unknown distribution parameters from [61] was used. For the gamma distribution, MATLAB code Fastfit [62] was used for parameter estimation. For the Pareto distribution, known closed-form solutions for ML estimators are used.

### L-moment ratio diagram

The l-moment ratio diagram [63,64] of l-skewness verus l-kurtosis was created using the "lmomc" package [65] in the R statistical software [66].

### Mixture distribution fit

The "Male" and "Female" data were fitted to mixture of normal distributions using the "mixdist" package [67] in the R statistical package [66].

## Results

The Kruskal Wallis test was used on the logarithm of expression of the probe sets in each of the "Craniofacial", "Liver" and "Brain" data sets in order to identify those that were most likely unaffected by any of the conditions involved in the generation of these data sets. The results in Table 2 indicate that more than 50% (~25000/45000) of the probe sets were unaffected in the "Brain" data set while a far fewer were unaffected for the "Craniofacial" and the "Liver" data sets. Further the results in Table 2 indicate that of these unaffected probe sets for each of the three data sets, more than 50% rejected the null hypotheses (at a 95% confidence level) for all the distribution tests considered - Normal, Weibull, Extreme value, Logistic, Log-normal and Log-logistic. The same can be said about the house keeping genes [47]. The data for these are based on 6219 microarray samples. Also, it seems that at least one of the above six distributions failed to be rejected for between 25 and 52% of the probe sets in each of the three data sets. Additional simulation-based Anderson-Darling (AD) tests were performed for the Gamma and Pareto distributions for the house keeping genes. 23/23 housekeeping probe sets rejected the AD null hypothesis for the Gamma distribution at the 95% confidence level while only 2/23 rejected the hypotheses for the Pareto distribution. However, the Kolmogorov-Smirnov (KS) test rejected 23/23 null hypotheses for both the Gamma and Pareto distributions. Additional results obtained by varying the Kruskal Wallis test p-value cutoff and the results obtained from the Kolmogorov-Smirnov goodness of fits tests are given in Additional files 5 and 6, Tables S6 and S4 respectively. Detailed probe level results are provided in Additional file 7, Table S5.

**Table 2:** Fraction of null hypotheses rejected by the Anderson-Darling tests for best fit to 7 distribution functions.

*Dataset*	Probe set no.	Normal	Weibull	Extreme Value	Logistic	Lognormal	Log-logistic	At least one of the distributions not rejected
*Craniofacial*	6215	0.72	0.79	0.82	0.69	0.75	0.71	0.46

*Liver*	6228	0.82	0.93	0.95	0.79	0.83	0.8	0.25

*Brain*	25146	0.77	0.92	0.93	0.69	0.77	0.68	0.35

*Housekeeping*	23	0.7	1	1	1	0.48	1	0.52

*Male*	19532	0.46	NA	0.96	0.24	NA	NA	0.82

*Female*	18915	0.43	NA	0.96	0.21	NA	NA	0.85

The fraction is the number of probe sets that reject a given hypothesis out of the number of the probe sets (that is given in the second column). The number of probe sets in the second column were the ones that were assumed to be unaffected by the conditions involved in the generation of all samples in each of the six data sets - "Craniofacial", "Liver", "Brain", "Housekeeping", "Male" and "Female". Probe sets were deemed to be unaffected for the first three data sets using the Kruskal Wallis test as described in the Methods section. The "Housekeeping" data set had 6219 samples and the 23 probe sets analyzed corresponding to the so-called housekeeping genes [47] that are supposed to be essential for cell-survival under most conditions. The probesets for the "Male" and "Female" data sets were identified using the procedure detailed in the Methods section. Note some of the log ratio data for the "Male" and "Female" data sets are negative and so cannot be tested for goodness-of-fit to some of the distributions. The results for these distributions are listed as "NA".

The results of the goodness of fit Anderson-Darling distribution tests for the "Male" and "Female" data sets showed different characteristics from those of the other data sets. Only around 43-46% of the probe sets rejected the normal hypothesis (as compared with 72-82% for the previous data sets). The logistic distribution was rejected less often than the previous data sets (21-24% as compared with 69-79%). The fit of the extreme value distributions were equally bad for both sets of data. This difference in characteristics of the goodness of fit test results between two different microarray platforms indicates the contribution of technology and/or of the normalization methodology to the distribution characteristics of microarray data.

**Figure 1:**
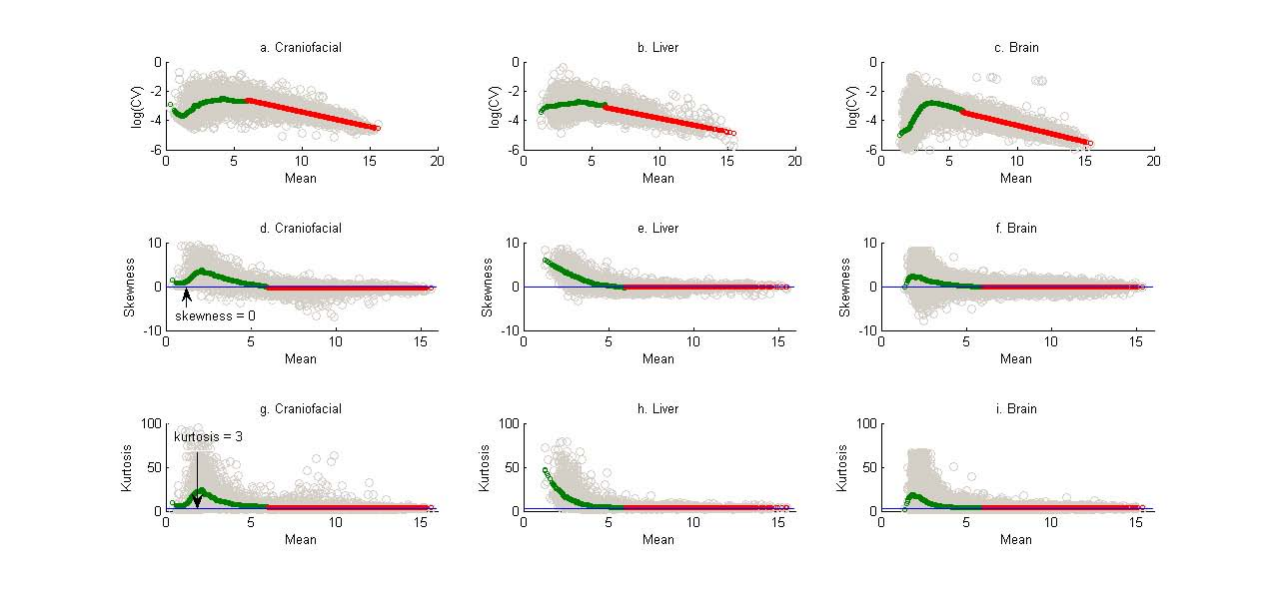
**Scatter of mean versus coefficient of variation, skewness and kurtosis of distribution of gene expressions for the three Affymetrix data sets**. The green curves in each of the plots represent the loess curves for scatter of each of the three higher moments (coefficient of variation, skewness and kurtosis) versus the mean of the gene expression for all those genes whose mean is less than 6. The red curves represent the best fit polynomials for scatter of the higher moments versus the mean for all those genes or probe sets whose gene expression is greater than 6. The fitted trend is linear between the logarithm of the coefficient of variation and the mean of the distribution of the log of gene expressions and constant for the relationships between skewness and mean and between kurtosis and mean.

**Figure 2:**
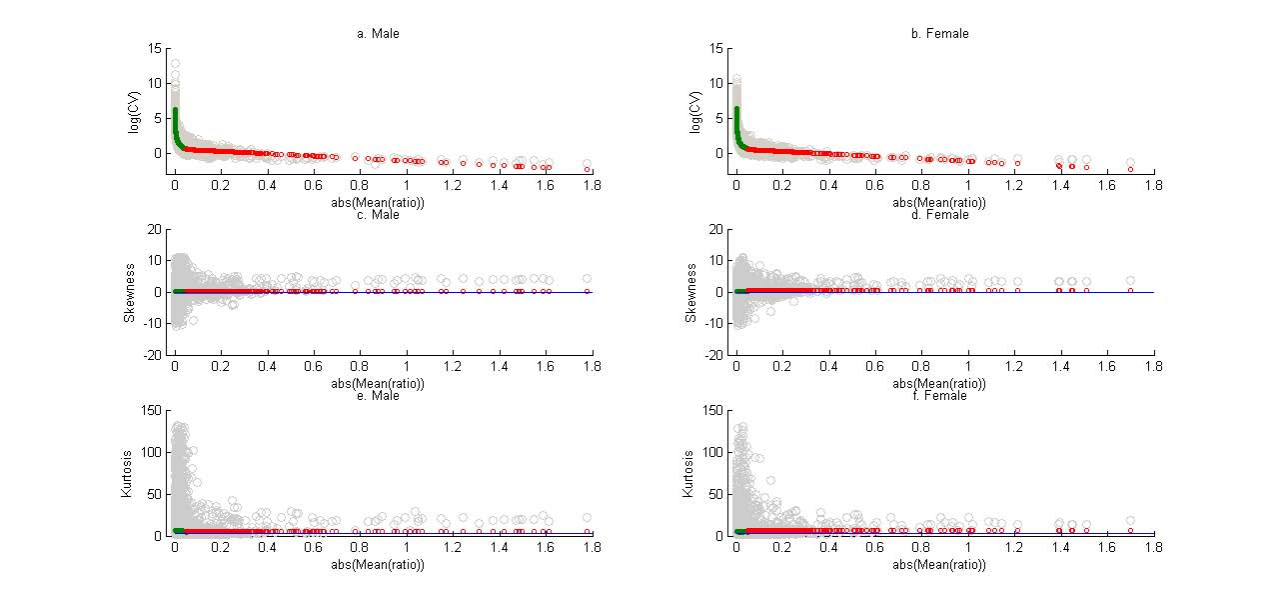
**Scatter of mean log ratio versus coefficient of variation, skewness and kurtosis of distribution of gene expressions for the two Rosetta/Merck data sets**. The green curves in each of the plots represent the loess curves for scatter of each of the three higher moments (coefficient of variation, skewness and kurtosis) versus the mean log ratio of the gene expression for all those genes whose absolute value is less than 0.05. The red curves represent the best fit polynomials for scatter of the higher moments versus the mean for all those genes or probe sets whose absolute value of the mean log ratio of gene expression is greater than 0.05. The fitted trend is linear between the logarithm of the coefficient of variation and the mean of the distribution of the log of gene expression ratio and constant for the relationships between skewness and mean and between kurtosis and mean.

The dependence of the mean on the higher order product moments are shown in Figures 1 and 2 - 1(a)-1(c), 2(a)-2(b): (mean versus coefficient of variation), 1(d)-1(f), 2(c)-2(d): (mean versus skewness) and 1(g)-1(i), 2(e)-2(f): (mean versus kurtosis). Note in the case of the "Male" and "Female" data sets the higher order moments are plotted against the absolute value of the mean of the log-ratio data. The overall trends of the higher moments with the means are consistent. Note that in the range of lower mean expression the coefficient of variation, skewness and kurtosis are unusually high. This probably reflects genes whose expressions are below the detection limit of the microarray and can be treated as optical noise [29] for the case of the Affymetrix data. For the "Male" and "Female" data this may reflect the instability of the ratio for low expression genes. By visual inspection of Figure 1, a value of 6 is chosen as cutoff of mean of the logarithm of gene expression so that mean of the logarithm of the measurements of the gene expression below 6 are considered noise and a cutoff of 0.05 absolute value of ratio was chosen for Figure 2. Loess curves (represented by green lines in the subplots) are fit through the noisy measurements and polynomials (represented by red lines in the subplots) are fit through the other data to describe the trend between mean and the next three higher product moments. A linear fit to the trend between the logarithms of the coefficient of variation (CV) and the mean of the logarithms of mean of expression of different genes are shown in Figure 1. The parameters of the linear fit are given in Table 3. The decreasing trend of the coefficient of variation with the mean of gene expression is valid across the five data sets. This recapitulates what is already known about microarray data (see [35]). The suggestion of consistency between the trends is also clear from observation of the coefficients in Table 3 (separately for the Affymetrix data sets and the Rosetta/Merck ("Male", "Female") data sets). This consistency is however not statistically valid. Also, note that the confidence intervals generated for the parameters in Table 3 are probably not entirely valid given issues of heteroscedasticity and non-normality of the residuals associated with the three fits (see Additional files 8, 9, 10, Figures S1-S3). Skewness and kurtosis did not have a significant trend with the mean of distribution the gene expression as measured by the R^2 ^values for different polynomial fits (the R^2 ^values did not exceed 0.1). So the mean values of skewness and kurtosis are plotted by the red lines in Figure 1d-1i, 2c-2f. These mean values are given in Table 4. The Normal distribution has a skewness value of 0 and a kurtosis of 3. The values in Table 4 suggest a majority of the distributions of gene expression as being more negatively (positively) skewed than the Normal and as having a higher (higher) kurtosis value for the Affymetrix (Rosetta/Merck) data.

**Table 3:** Coefficients of the linear trends between the logarithm of the coefficient of variation (CV) and the mean of the distribution of the logarithm of gene expression for each of the three data sets.

*Data set*	Intercept	Slope	**R**^**2**^	p-value
*Craniofacial*	-1.38 ± 0.06	-0.20 ± 0.01	0.6	0

*Liver*	-1.99 ± 0.11	-0.19 ± 0.02	0.44	0

*Brain*	-2.10 ± 0.04	-0.22 ± 0.01	0.62	0

*Male*	0.57 ± 0.02	-1.67 ± 0.12	0.29	0

*Female*	0.56 ± 0.02	-1.74 ± 0.13	0.3	0

Also included in the table are the 95% confidence intervals for these coefficients, the coefficient of determination, R^2 ^and the p-value for the goodness of fit F-test to the linear model. Note that the models are computed using data over those probe sets with mean expression greater than 6 in order to avoid analysis with noisy or low expression genes.

**Table 4:** Means of skewness and kurtosis of distribution of log of gene expression.

*Data set*	Mean skewness	Mean kurtosis
*Craniofacial*	-0.39 ± 0.03	4.02 ± 0.12

*Liver*	-0.16 ± 0.03	3.82 ± 0.08

*Brain*	-0.1 ± 0.02	3.52 ± 0.03

*Male*	0.26 ± 0.05	5.20 ± 0.31

*Female*	0.40 ± 0.06	5.97 ± 0.38

Also included is the 95% confidence interval for these means. Note that the means are computed over those probe sets with mean expression greater than 6 in order to avoid analysis with noisy or low expression genes.

**Figure 3:**
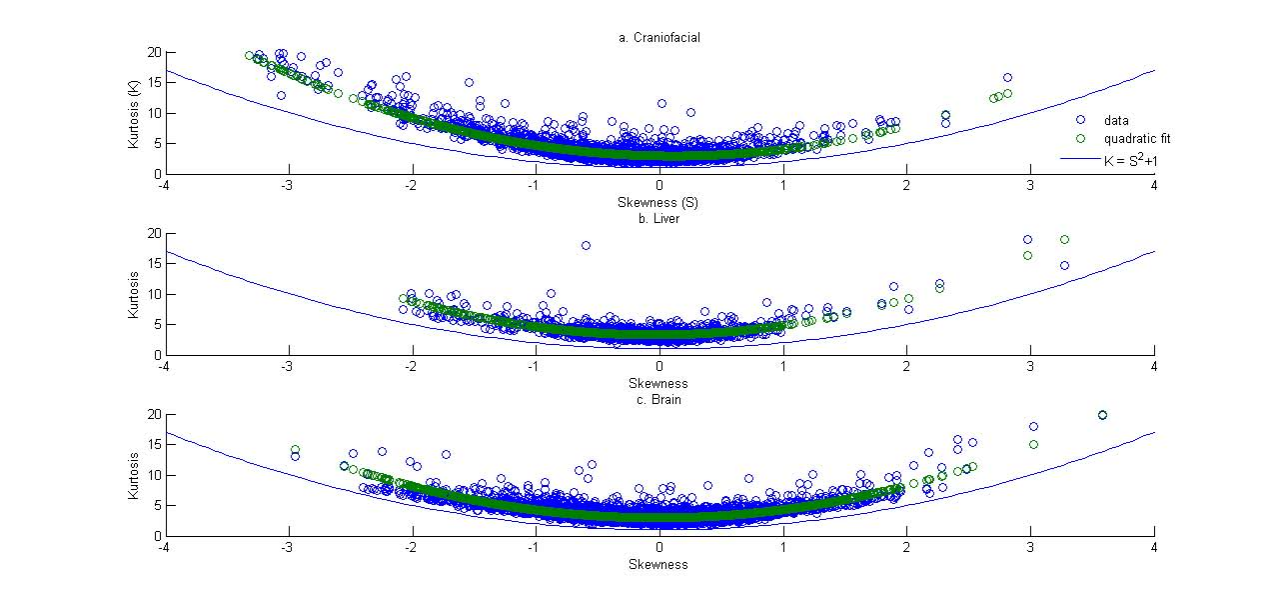
**Scatter of kurtosis versus skewness and of distribution of gene expressions for the three data sets - "Craniofacial" in (a) "Liver" in (b) and "Brain" in (c)**. The green circles represent the fit using the best fit quadratic polynomial. The blue line represents the theoretical limit of the scatter between the skewness and the kurtosis of a probability distribution.

**Figure 4:**
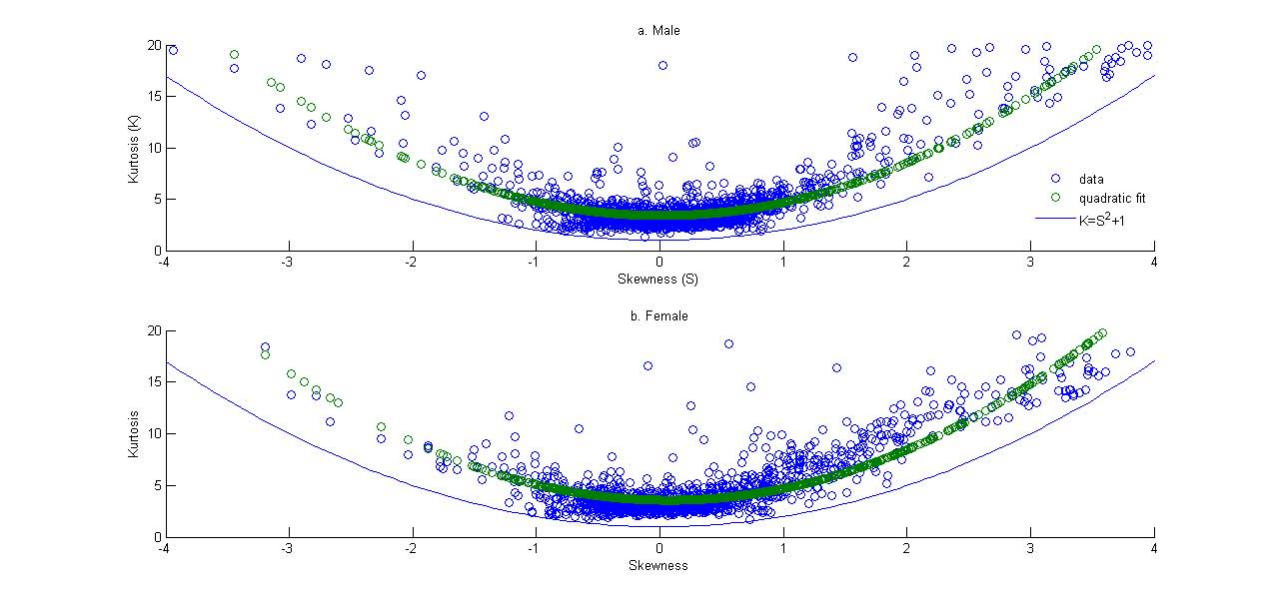
**Scatter of kurtosis versus skewness and of distribution of gene expressions for the two data sets - "Male" in (a) and "Female" in (b)**. The green circles represent the fit using the best fit quadratic polynomial. The blue line represents the theoretical limit of the scatter between the skewness and the kurtosis of a probability distribution.

The relationship between the kurtosis and the skewness of the distribution of gene expressions is studied next. The variations of kurtosis of the distribution of gene expression with skewness for the three data sets are plotted in Figures 3 and 4. Note this plot is restricted to only those genes with mean expression greater than 6 for Figure 3 and absolute value log ratio greater than 0.05 for Figure 4. The quadratic fits to the trends are also shown in Figures 3 and 4. The coefficients of the fit are given in Table 5. The confidence intervals for these coefficients may again be questioned given the issues with the heteroscedasticity and non-normality of the residuals (see Additional files 11, 12, 13, Figures S4-S6). Alternate non-linear models were evaluated for their ability to alleviate these issues. However a solution wasn't found that would make reduce both concerns simultaneously. So in the interest of simplicity and also given the fact the stated confidence intervals are not used to draw further conclusions, the results are presented without making any further attempts to satisfy the assumptions of homoscedasticity and normality of residuals. Theoretically, the kurtosis (denoted by *K*) and the skewness (denoted by *S*) have to satisfy the following inequality (see [68]),

$$K\geq S^{2}+1$$

**Table 5:** Coefficients of the quadratic trends between the kurtosis and skewness of the distribution of the logarithm of gene expression for each of the three data sets.

*Data set*	Constant	Linear	Quadratic	**R**^**2**^	p-value
*Craniofacial*	2.96 ± 0.04	-0.31 ± 0.07	1.40 ± 0.03	0.91	0

*Liver*	3.30 ± 0.07	0.12 ± 0.10	1.42 ± 0.06	0.58	0

*Brain*	2.97 ± 0.03	0.03 ± 0.03	1.31 ± 0.03	0.65	0

*Male*	3.65 ± 0.34	-0.05 ± 0.27	1.21 ± 0.07	0.82	0

*Female*	3.85 ± 0.27	-0.12 ± 0.20	1.23 ± 0.05	0.89	0

Also included in the table are the 95% confidence intervals for these coefficients, the coefficient of determination, R^2 ^and the p-value for the goodness of fit F-test to the linear model. Note that the models are computed using data over those probe sets with mean expression greater than 6 in order to avoid analysis with noisy or low expression genes.

The data in Table 5 are again suggestive (though not statistically valid) of a consistent quadratic trend across the three data sets between kurtosis and skewness of the distributions of gene expression.

**Figure 5:**
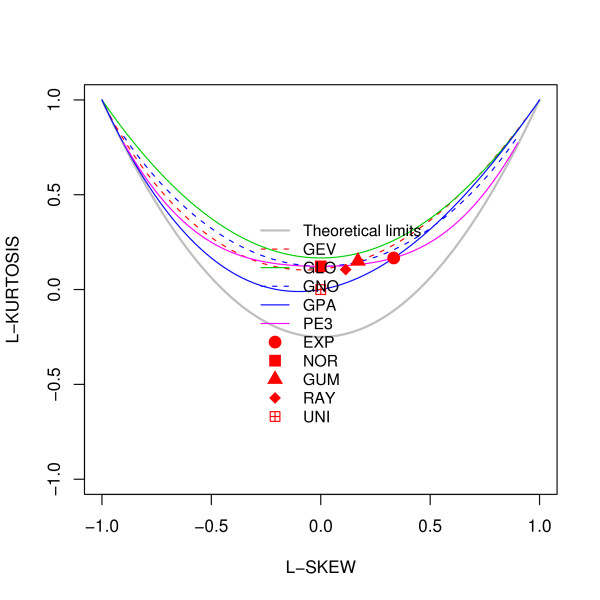
**Legend for the theoretical probability distributions in the l-moment ratio diagrams provided as subplots in Figure 2**. GEV - Generalized Extreme Value, GLO - Generalized Logistic, GNO - Generalized Normal, GPA - Generalized Pareto, PE3 - Pearson Type III, EXP - Exponential, NOR - Normal, GUM - Gumbel, RAY - Rayleigh and UNI - Uniform.

**Figure 6:**
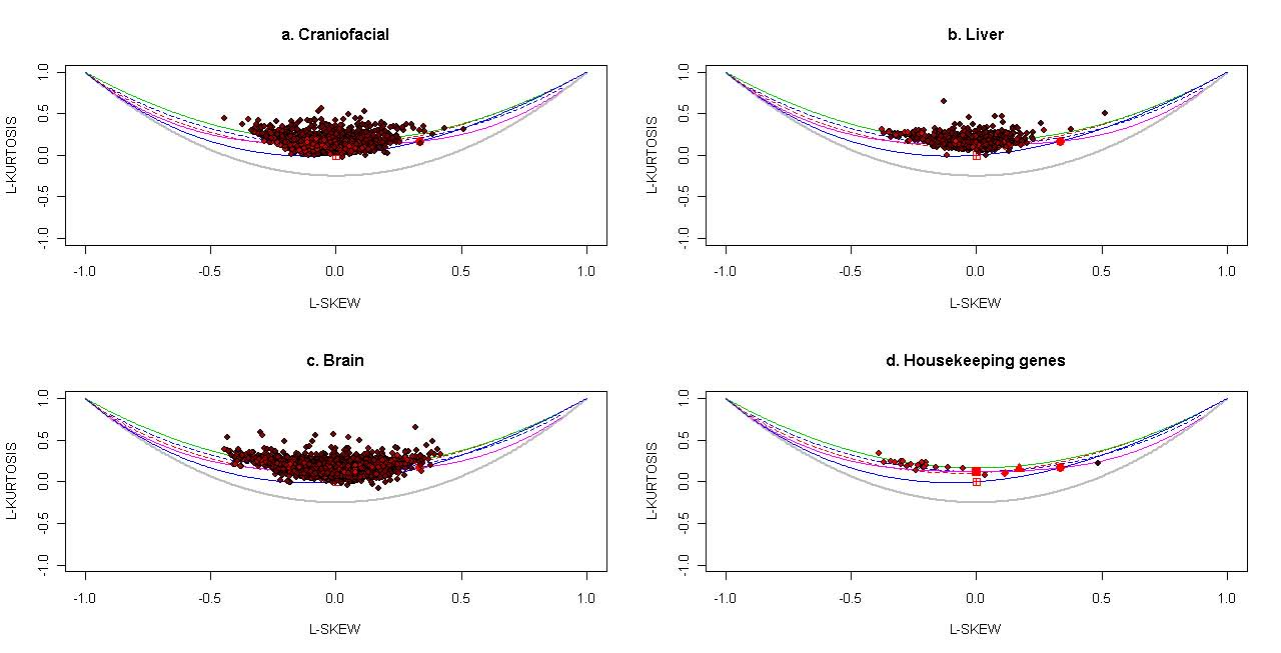
**Scatter of l-skewness versus l-kurtosis of gene expressions for the four data sets - "Craniofacial", "Liver", "Brain" and "Housekeeping"**. The scatter is overlaid on the relationships between these moments for standard theoretical distributions. The legend for these distributions is provided in Figure 5. The l-skewness and l-kurtosis for the unaffected probe sets with mean expression greater than 6 for the "Craniofacial", "Liver", "Brain" and "Housekeeping" datasets are plotted in the subplots (a), (b), (c) and (d) respectively. The intensity of red of each circle that represents a probe set in these subplots is made to vary linearly with the mean expression of this probe set. So higher the mean expression, the greater is the intensity of red.

**Figure 7:**
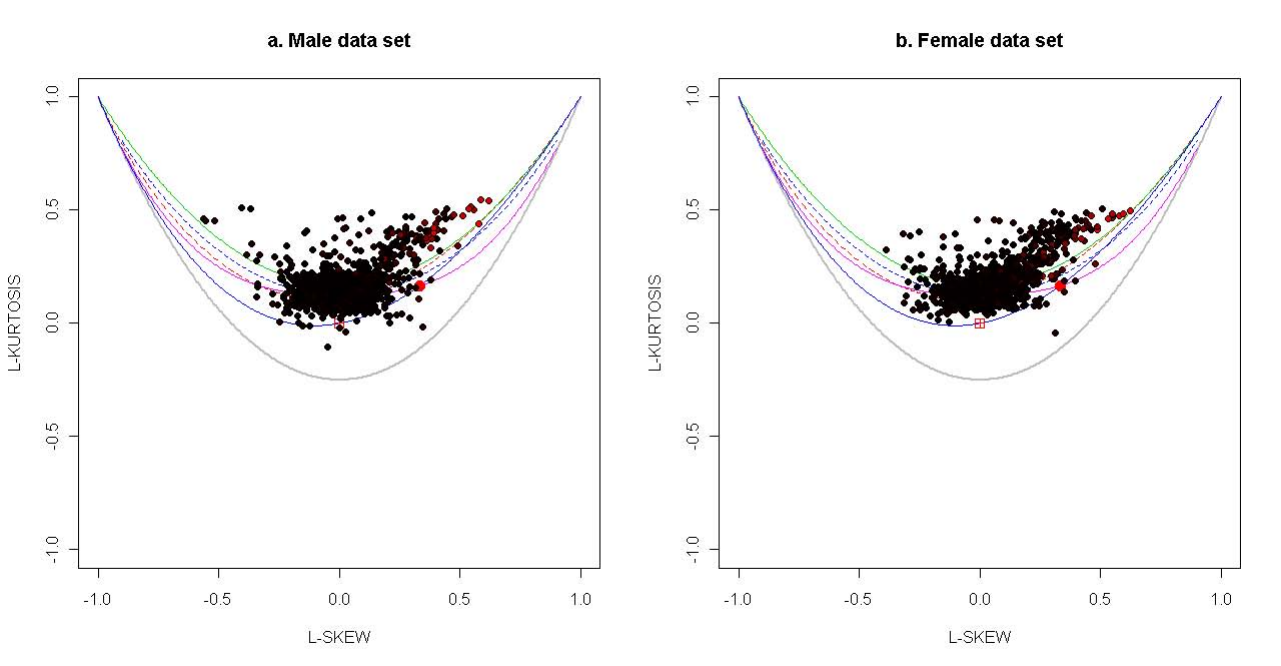
**Scatter of l-skewness versus l-kurtosis of gene expressions for the two Rosetta/Merck data sets - "Male" and "Female"**. The scatter is overlaid on the relationships between these moments for standard theoretical distributions. The legend for these distributions is provided in Figure 5. The l-skewness and l-kurtosis for the unaffected probe sets with mean log ratio of expression greater than 0.05 for the "Male" and "Female" datasets are plotted in the subplots (a) and (b) respectively. The intensity of red of each circle that represents a probe set in these subplots is made to vary linearly with the mean expression of this probe set. So higher the mean expression, the greater is the intensity of red.

Further validation of the lack of fit of gene expression to any of the standard theoretical univariate probability distributions can be seen in the L-moment ratio diagram in Figure 5. The theory of L-moments was introduced in [63] and they have been shown to provide unbiased estimates of the higher order moments of a probability distribution. The standard product-moments have been shown to be significantly affected by the sample size and the presence of outliers [64]. The relationship between the third-order and fourth-order L-moments (representing the skewness and kurtosis of the distribution) can be plotted on an diagram called the L-moment ratio diagram. This is shown in Figure 5. The L-skewness and L-kurtosis for all the unaffected probe sets (with mean expression greater than 6) in each of four Affymetrix data sets are plotted in Figure 6a-6d and for the actively expressed genes (with absolute value of log ratio greater than 0.05) for the Rosetta/Merck data sets in Figure 7a-7b. The l-moment plots with all the data including those with mean expression less than 6 for the Affymetrix data is provided in Additional file 14, Figure S7 and those for all Rosetta/Merck data including those with absolute value of mean of log ratio of gene expression greater than 0.05 is provided in Additional file 15, Figure S8. The probe sets in each of these subplots represented as circles are also colored. The intensity of redness of probe set circle reflects its mean expression. This coloring scheme points to the lack of dependence of the L-(skewness and kurtosis) moments on the mean expression. The consistent non-linear relationships across the three data sets between the product moments skewness and kurtosis identified in Figures 3 and 4 also appear to hold for the l-skewness and l-kurtosis.

The samples from the "Male" and "Female" datasets could be considered more or less homogenous with respect to sex, tissue, diet and experimental conditions. One reason we could be seeing poor fits to standard distributions could be that there are different modes to the distribution of the expression of a given gene reflecting the genetic variation in the F2 cross animals or a stochastic network influence (e.g. gene involved in a given biochemical pathway) of its expression [24]. Hence it is possible for a mixture of distributions to better fit the observed data. The results of chi-square fits to a three normal mixture distribution are given in Table 6. There is no improvement in fit to the mixture distribution on comparison of the results with the chi-square fit to a single normal.

**Table 6:** Fraction of null hypotheses rejected by the Chi-square test for the goodness-of-fit to a normal distribution and a mixture of three normal distributions for the "Male" and "Female" data sets

Data set	Normal	3-normal mixture
*Male*	0.18	0.17

*Female*	0.16	0.17

## Discussion

In the past several years, there has been an explosion in amount of quantitative biological data either in terms of transcriptomics, sequencing data, genetic structure variation, proteomics or metabolomics. DNA microarrays have been important and valuable resource for understanding perturbations to biological systems in terms of identifying affected gene expressions. The standard statistical methods are being either directly used or modified to work with gene expression data. Unfortunately, only a small number of replicate samples per treatment (2-10) are used for analysis owing to the cost of the experimentsal system. This point plus the fact that the probability distributions of gene expressions as measured by these arrays were not characterized leads one of logically question the use various statistical methods that are based on distributional assumptions. Heuristics are being proposed that attempt to relax the reliance on this distributional assumption. One example of this would be the method of jointly using the p-value from a two sample t-test along with the gene expression fold-change to identify differentially expressed genes. Alternately, there is an increased use of non-parametric methods or permutation-based methods [69,38].

The essential question that we address in this manuscript is whether the distribution of the logarithm of gene expression as measured by DNA microarrays can be approximated by any of the standard theoretical univariate probability distributions. The results in Table 2 and Figures 6 and 7 suggest that it is unlikely that there is a known probability distribution that all gene expressions would follow. Now if this is the case then an alternative would be is to see if there are consistent relationships between the various moments of the distributions of the gene expressions. The analyses in Figure 1, 2, 3, 4 suggest that there are consistent (though not statistically valid) relationships between the mean and coefficient of variation (Additional file 16, Table S7 lists the spearman rank correlation between the mean and standard deviation of the gene expressions corresponding to each of the data sets analyzed) and the skewness and kurtosis of the distribution of gene expression respectively. Comparison of results as obtained from a commercial Affymetrix platform and a non-comercial Rosetta/Merck platform also indicated the influences of the microarray technology on the distribution characteristics of the data.

The observed distributional characteristics of gene expression data in this manuscript suggest either the need for the use of non-parametric statistical methods or a need to develop newer statistical/mathematical approaches that are capable of and are optimal for working with these kinds of distributions.

Because of the nature of the data used in this paper, we are unable to separate the contribution to the variation of the data due to biological reasons from those induced by the microarray technology. The noise we observe is probably the result of the convolution of these two factors. In light of the increasing use of newer technologies like those based on Next Generation Sequencing [70] and despite the real possibility of the reduced future use of DNA microarrays an analysis like that presented in this paper would be useful in guiding analyses of these new data and also in making distributional hypotheses.

## Conclusions

The analyses of the empirical probability distribution of gene expressions from five publicly available data sources with relatively large number of samples have been described in this manuscript. The failure of the distributions to follow any of the known theoretical univariate probability distributions has been demonstrated though the data suggests consistent relationships between the different moments of the distributions. These moment relationships should motivate the development of Bayesian methods with appropriately chosen priors.

## Authors' contributions

RT and SM designed the study. LdT carried out the all goodness of fits analyses and contributed to the write-up. XC contributed in checking the annotations of the entire microarray data samples used and also in the interpretation of the results. RT performed the l-moment analysis and drafted the manuscript. SM also contributed to the drafting of the manuscript. All authors read and approved the final manuscript.

## Supplementary Material

Additional file 1 **Table S8**: Detailed description of the data in Table 1. This includes more description of the microarray platform and the base pair length of the probes used on each of the microarray platforms. Click here for file

Additional file 2 **Table S3**: List of housekeeping genes [47] analyzed Click here for file

Additional file 3 **Table S1**: GEO [30] microarray samples for the "Craniofacial", "Liver" and "Brain" data sets. Click here for file

Additional file 4 **Table S2**: GEO [30] microarray samples used to analyze the expression of probe sets corresponding to the house keeping genes Click here for file

Additional file 5 **Table S6**: Results of goodness of fit tests for the Anderson-Darling tests for the six analyzed probability distributions obtained by the varying the cutoff for the Kruskal-Wallis test. Click here for file

Additional file 6 **Table S4**: Best fit distribution Kolmogorov-Smirnov test results for "Craniofacial", "Liver", "Brain", "Male" and "Female" data sets Click here for file

Additional file 7 **Table S5**: Probe set level best fit distribution results for the Anderson Darling (AD) and the Kolmogorov-Smirnov test (KS) tests at 90 and 95 percent confidence levels Click here for file

Additional file 8 **Figure S1**: Diagnostic plot for the linear model between the logarithm of the coefficient of variation (CV) and the mean of the distribution of the logarithm of gene expression for the "Craniofacial" data set. Note the residual plots (subplots (b) and (c)) also provide the pearson correlation (denoted by ρ) between the absolute value of the residuals and the mean and logarithm of the CV respectively. Click here for file

Additional file 9 **Figure S2**: Diagnostic plot for the linear model between the logarithm of the coefficient of variation (CV) and the mean of the distribution of the logarithm of gene expression for the "Liver" data set. Note the residual plots (subplots (b) and (c)) also provide the pearson correlation (denoted by ρ) between the absolute value of the residuals and the mean and logarithm of the CV respectively. Click here for file

Additional file 10 **Figure S3**: Diagnostic plot for the linear model between the logarithm of the coefficient of variation (CV) and the mean of the distribution of the logarithm of gene expression for the "Brain" data set. Note the residual plots (subplots (b) and (c)) also provide the pearson correlation (denoted by ρ) between the absolute value of the residuals and the mean and logarithm of the CV respectively. Click here for file

Additional file 11 **Figure S4**: Diagnostic plot for the quadratic model between the kurtosis and the skewness of the distribution of the logarithm of gene expression for the "Craniofacial" data set. Note the residual plots (subplots (b) and (c)) also provide the pearson correlation (denoted by ρ) between the absolute value of the residuals and the skewness and kurtosis respectively. Click here for file

Additional file 12 **Figure S5**: Diagnostic plot for the quadratic model between the kurtosis and the skewness of the distribution of the logarithm of gene expression for the "Liver" data set. Note the residual plots (subplots (b) and (c)) also provide the pearson correlation (denoted by ρ) between the absolute value of the residuals and the skewness and kurtosis respectively. Click here for file

Additional file 13 **Figure S6**: Diagnostic plot for the quadratic model between the kurtosis and the skewness of the distribution of the logarithm of gene expression for the "Brain" data set. Note the residual plots (subplots (b) and (c)) also provide the pearson correlation (denoted by ρ) between the absolute value of the residuals and the skewness and kurtosis respectively. Click here for file

Additional file 14 **Figure S7**: l-moment ratio diagram for the four data sets as in Figure 6 but including all probe sets including those with mean expression less than 6. Click here for file

Additional file 15 **Figure S8**: l-moment ratio diagram for the four data sets as in Figure 7 but including all probe sets including those with absolute value of mean of log expression ratio less than 0.05. Click here for file

Additional file 16 **Table S7**: Spearman rank correlation between the mean and standard deviation of the measured data for "Craniofacial", "Liver", "Brain", "Male" and "Female" data sets. Click here for file
