# Supplementary figures and images for: Validation and characterization of DNA microarray gene expression data distribution and associated moments
Source: BMC Bioinformatics. 2010 Nov 24;11:576. doi: 10.1186/1471-2105-11-576 (PMC3002903; doi:10.1186/1471-2105-11-576)

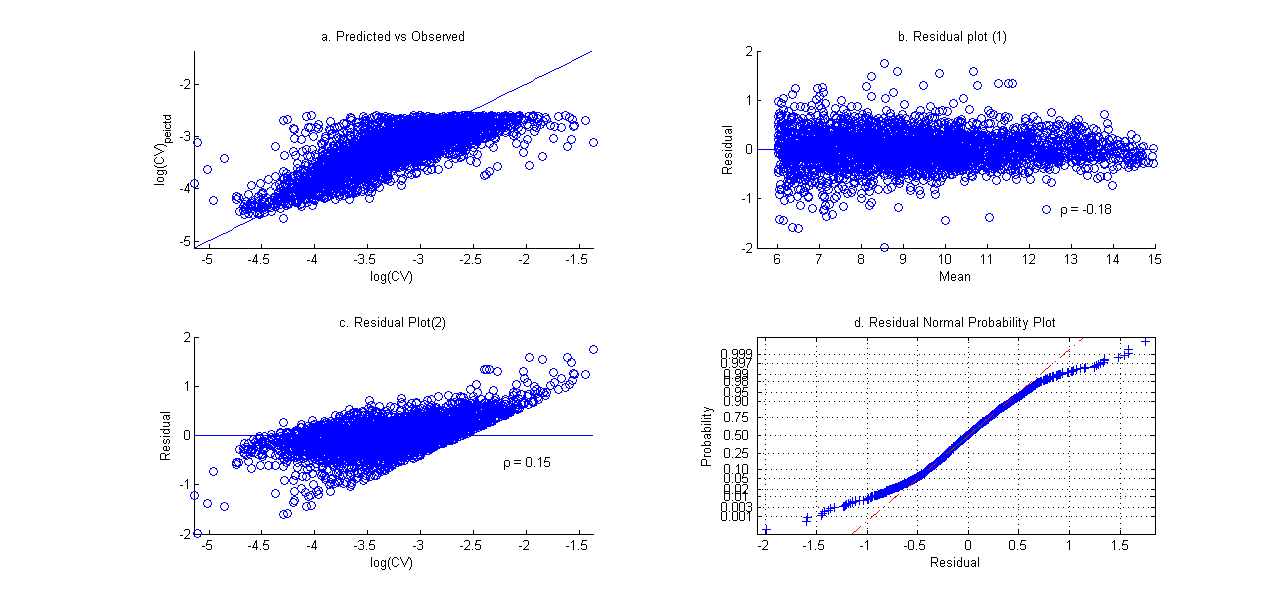

Supplement: Additional file 8 — Figure S1: Diagnostic plot for the linear model between the logarithm of the coefficient of variation (CV) and the mean of the distribution of the logarithm of gene expression for the "Craniofacial" data set. Note the residual plots (subplots (b) and (c)) also provide the pearson correlation (denoted by ρ) between the absolute value of the residuals and the mean and logarithm of the CV respectively. [file 1471-2105-11-576-S8.TIFF]

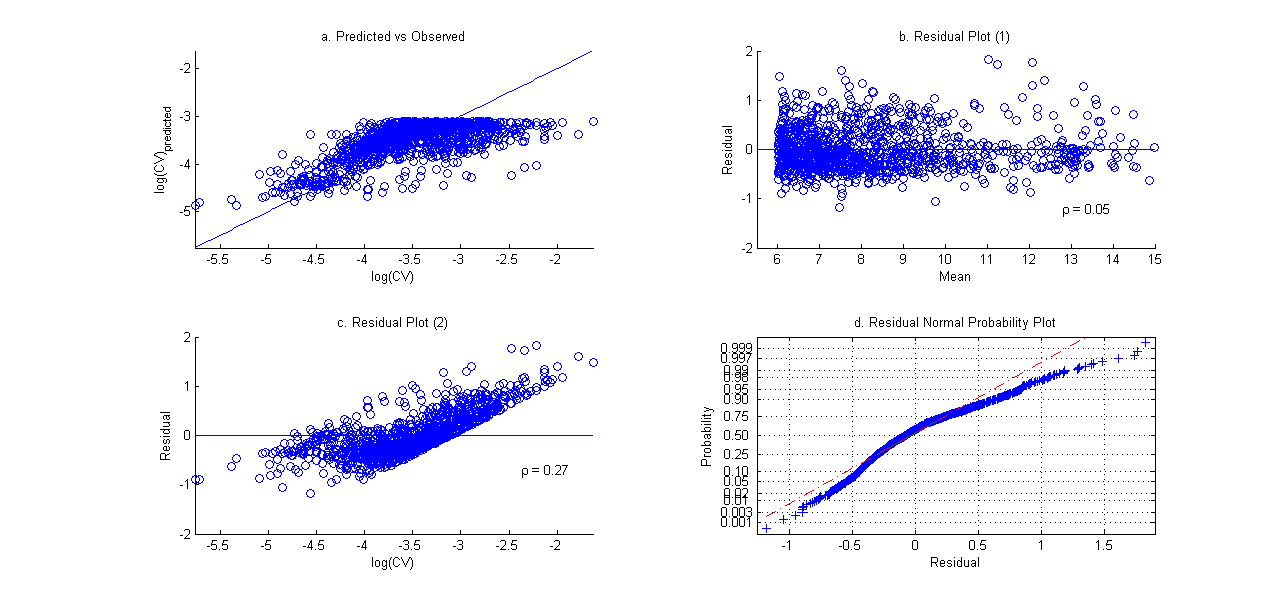

Supplement: Additional file 9 — Figure S2: Diagnostic plot for the linear model between the logarithm of the coefficient of variation (CV) and the mean of the distribution of the logarithm of gene expression for the "Liver" data set. Note the residual plots (subplots (b) and (c)) also provide the pearson correlation (denoted by ρ) between the absolute value of the residuals and the mean and logarithm of the CV respectively. [file 1471-2105-11-576-S9.TIFF]

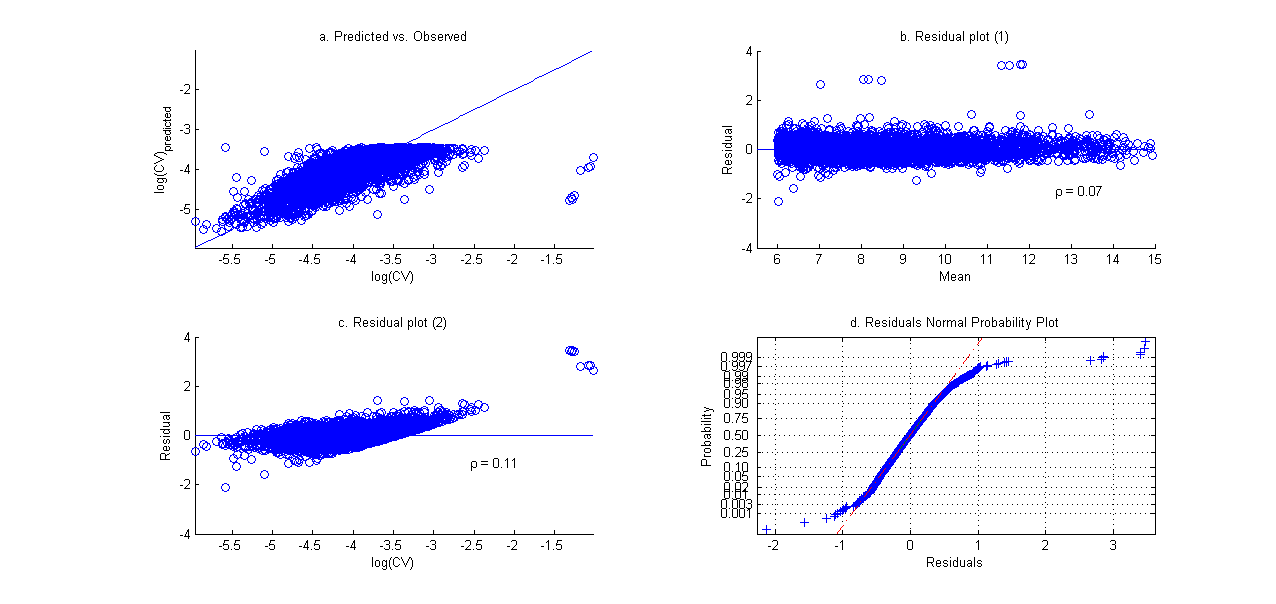

Supplement: Additional file 10 — Figure S3: Diagnostic plot for the linear model between the logarithm of the coefficient of variation (CV) and the mean of the distribution of the logarithm of gene expression for the "Brain" data set. Note the residual plots (subplots (b) and (c)) also provide the pearson correlation (denoted by ρ) between the absolute value of the residuals and the mean and logarithm of the CV respectively. [file 1471-2105-11-576-S10.TIFF]

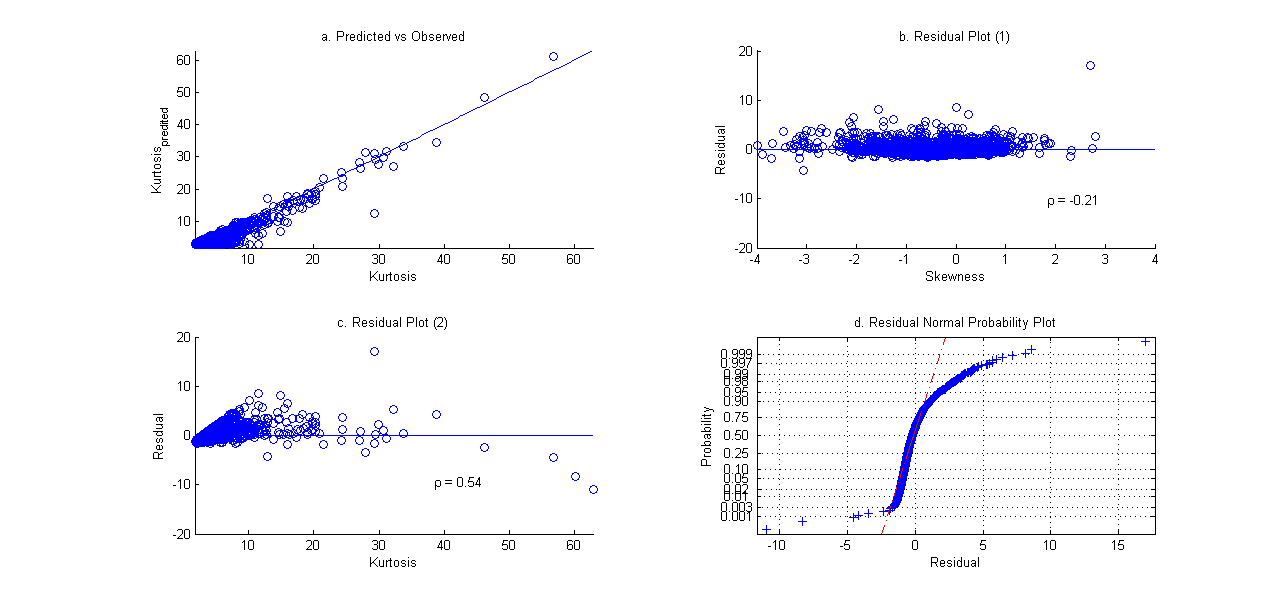

Supplement: Additional file 11 — Figure S4: Diagnostic plot for the quadratic model between the kurtosis and the skewness of the distribution of the logarithm of gene expression for the "Craniofacial" data set. Note the residual plots (subplots (b) and (c)) also provide the pearson correlation (denoted by ρ) between the absolute value of the residuals and the skewness and kurtosis respectively. [file 1471-2105-11-576-S11.TIFF]

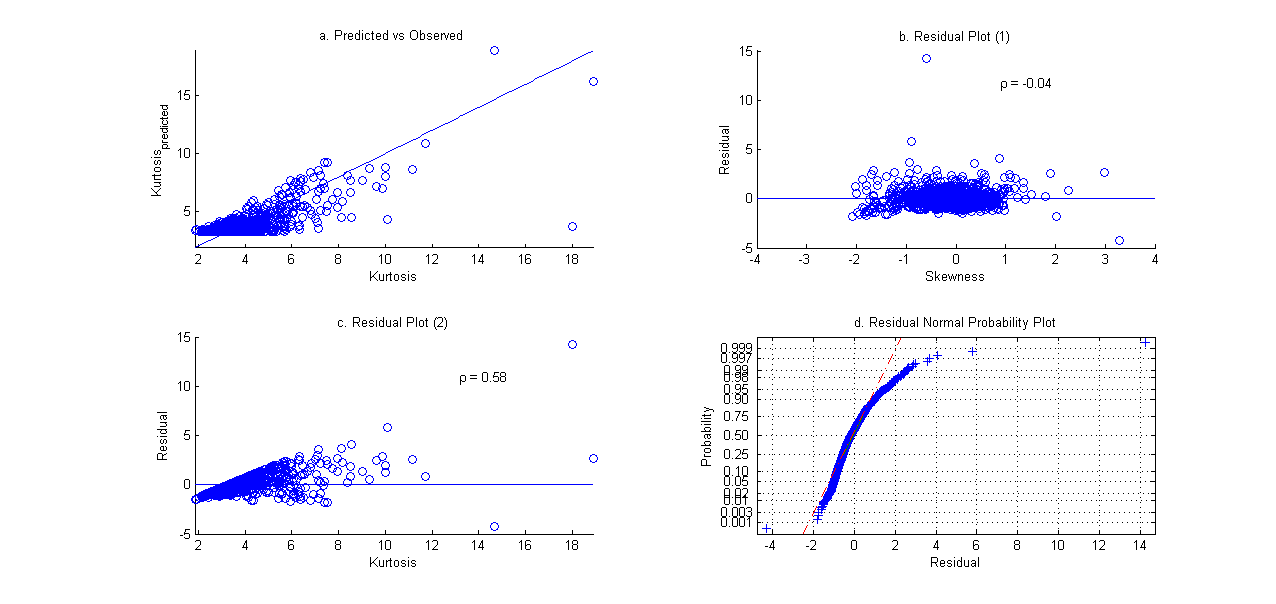

Supplement: Additional file 12 — Figure S5: Diagnostic plot for the quadratic model between the kurtosis and the skewness of the distribution of the logarithm of gene expression for the "Liver" data set. Note the residual plots (subplots (b) and (c)) also provide the pearson correlation (denoted by ρ) between the absolute value of the residuals and the skewness and kurtosis respectively. [file 1471-2105-11-576-S12.TIFF]

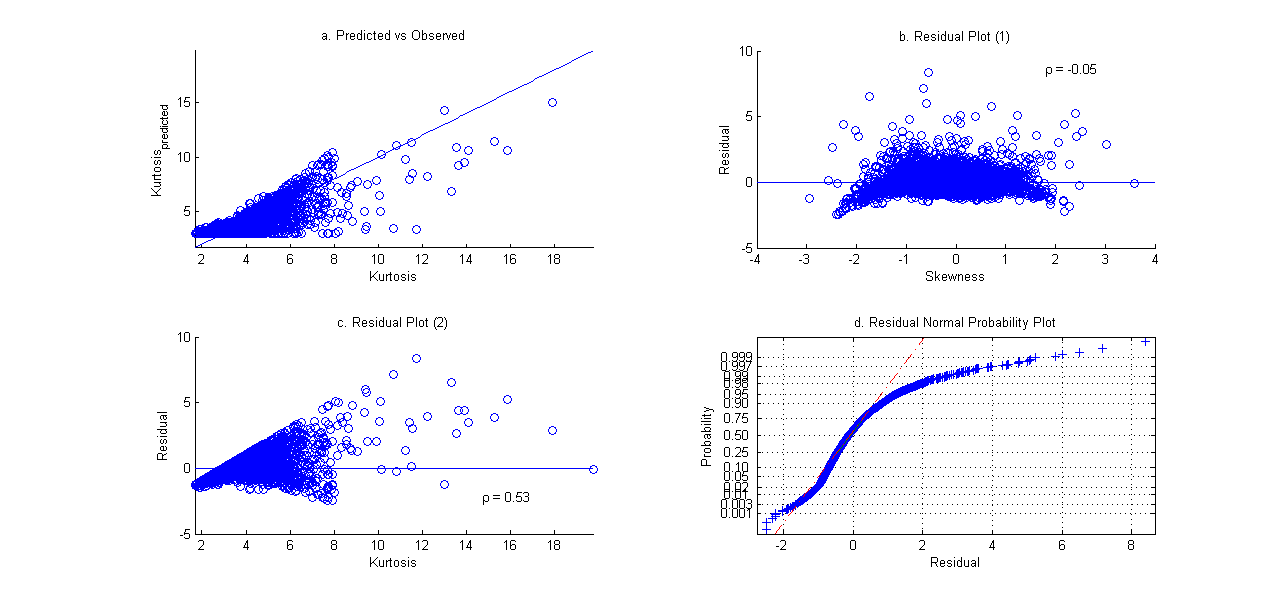

Supplement: Additional file 13 — Figure S6: Diagnostic plot for the quadratic model between the kurtosis and the skewness of the distribution of the logarithm of gene expression for the "Brain" data set. Note the residual plots (subplots (b) and (c)) also provide the pearson correlation (denoted by ρ) between the absolute value of the residuals and the skewness and kurtosis respectively. [file 1471-2105-11-576-S13.TIFF]
